# The hidden crisis: COVID-19 and impact on mental health of medical students in Pakistan

**DOI:** 10.1186/s43045-021-00123-7

**Published:** 2021-09-01

**Authors:** Nazish Imran, Imran Ijaz Haider, Ali Burhan Mustafa, Irum Aamer, Zahid Kamal, Ghulam Rasool, Muhammad Waqar Azeem, Afzal Javed

**Affiliations:** 1 Department of Child and Family Psychiatry, King Edward Medical University/Mayo Hospital, Lahore, Pakistan; 2 Fatima Memorial Hospital (FMH) College of Medicine & Dentistry, Lahore, Pakistan; 3 Sheikh Zayed Medical College/Hospital, Rahim Yar Khan, Pakistan; 4 Academic Department of Psychiatry and Behavioral Sciences, King Edward Medical University/Mayo Hospital, Lahore, Pakistan; 5 Sahiwal Medical College, Sahiwal, Pakistan; 6 Bolan Medical College, Quetta, Pakistan; 7 Baluchistan Institute of Psychiatry and Behavioural Sciences, Quetta, Pakistan; 8 College of Physicians and Surgeons Pakistan, Karachi, Pakistan; 9 Department of Psychiatry, Sidra Medicine, Weill Cornell Medicine-Qatar, Doha, Qatar; 10 Pakistan Psychiatric Research Centre, Fountain House, Lahore, Pakistan; 11 Institute of Applied Health Research, University of Birmingham, Birmingham, UK; 12 World Psychiatry Association, Geneva, Switzerland

**Keywords:** COIVD-19, Medical students, Mental health, Pakistan

## Abstract

**Background:**

Medical students have faced an enormous disruption to their lives due to the COVID-19 pandemic. The study aimed to assess the impact of COVID-19 on medical student’s psychological well-being in Pakistan. Following ethical approval, an online survey developed in collaboration with World Psychiatric Association (WPA) was distributed among medical students of 5 Medical colleges in the Punjab province of Pakistan between August and September 2020. Patient Health Questionnaire (PHQ-9), Generalized Anxiety Disorder Scale (GAD-7), and Risk Assessment Suicidality Scale (RASS) were used to assess psychological well-being. Data was analyzed using SPSS 26.0.

**Results:**

Eleven hundred medical students responded, 756 (69%) being females. More than 2/3rd admitted that their emotional state got worse in relation to appearance of anxiety, insecurity, and sadness, compared to before the outbreak of COVID-19. Prevalence of anxiety and depressive symptoms were 48.6% and 48.1%, respectively. Female medical students, pre-clinical students, and those with a previous psychiatric history reported experiencing more anxiety and depression symptoms (*P* value < 0.001). One in five medical students thought that it would be better if they were dead, and 8% admitted to often think of committing suicide during the past 2 weeks. RASS and subscales (intention, life, and history) scores were higher in females and students with previous psychiatric problems.

**Conclusion:**

Our findings underscore that the impact of COVID-19 on medical students has been significant; hence, it is crucial for medical colleges to employ strategies to maintain the student’s well-being with safeguards like reassurance, support, and confidential student-centered psychiatric services. The use of virtual platforms (websites, email) to educate and screen students by staff members can create a positive impact. The limitations of this study include cross-sectional design, the possibility of selective participation being web-based survey, response bias, and the possibility of reluctance of students to report mental health problems due to stigma.

## Background

High prevalence of mental health issues among medical students was already a public health concern prior to the COVID-19 pandemic with 27.2% and 11.1% of medical students from 167 countries reporting depressive symptoms and suicidal ideation in a meta-analysis [1]. As many as 33.8% reported anxiety symptoms [2]. Psychological distress among medical students deserves special attention. Depression and anxiety not only affect medical students lives in terms of their academic performance and professional development, impaired attention concentration and working memory, and increase risk of dropouts and poor quality of life, but also adversely affect patient care with more risk of clinical errors and having a less empathic attitude towards patients [3, 4].

In the fight against coronavirus disease, medical students have been particularly affected and are facing unprecedented challenges. Many factors including fear of infection, closure of medical colleges, widespread transition and adjustment to online learning, academic stress, abrupt cancelation of clinical postings, changes in assessments, and less social and academic support by friends and colleagues due to lockdown may be attributed towards increase vulnerability in medical students to depression and anxiety [5, 6]. In addition, for some students, living at homes in the absence of peer interactions and support may further have the potential to impact negatively on their well-being.

Many previous studies from Pakistan suggest a high prevalence of anxiety and depression among this population [7–9]. However, despite widespread concerns, to our knowledge, there are no published peer-reviewed articles on the effects of COVID-19 on medical students in Pakistan. With suggestions that the psychological impact of COVID-19 is likely to persist long after the pandemic is over, there is a pressing need to understand the psychological impact among medical students, so that effective mitigation strategies can be developed. Institutions also need to take steps to provide support to vulnerable students.

The aim of the current study was to provide a much-needed understanding of the mental health impacts of COVID-19 among medical students and provide new insight into which students may be most vulnerable for psychological distress due to this pandemic.

## Methods

This was a prospective observational multicenter study to collect real-time data about the mental health of medical students by disseminating an online questionnaire. Study protocol was approved by the Institutional Review Board of KEMU, and following permission, data was collected from five medical colleges (3 public sector and 2 private medical colleges) over 6 weeks commencing from 1st August 2020. All students enrolled in these institutions were eligible to participate. Students were approached via WhatsApp groups with an online survey link that the consenting participants could complete remotely via smartphone or computer. The questionnaire was anonymous and students were assured of the confidentiality of their responses. All students were given a 24-h contact number, as well as an e-mail of the principal author (NI) in case they were feeling distressed and needed emergency support. Details of several local resources and support groups were also provided.

The survey was co-developed with input from World Psychiatric Association (WPA) as well as medical students’ representatives and literature available on the topic. Feedback was sought from some local faculty members regarding its design, length, content, and usability. Based on their input, the questionnaire was updated to improve clarity, objectivity, and more accurately capture the needs of our participants during the COVID-19 pandemic.

The online survey questionnaire had various sections including demographic information; past history of psychiatric and medical illness; general state of health and well-being during COVID-19; thoughts about COVID and measures taken; questions about anxiety and depression and suicidal thoughts; impact on nutrition and physical activity, sleep, substance misuse, and learning; opinions about the origin of the epidemic and other health and public issues; and support offered to participants from their medical colleges. The current paper focuses on the mental health of the students during the COVID-19 pandemic.

### Assessment tools

#### Anxiety symptoms

The seven-item Generalized Anxiety Disorder (GAD-7; range 0–21) was used to assess anxiety symptoms. The severity of symptoms of anxiety is interpreted as normal (0–4), mild (5–9), moderate (10–14), and severe (15–21) anxiety [10]. GAD-7 has demonstrated strong psychometric properties and Cronbach’s alpha was 0.92 in the current study.

#### Depressive symptoms

To assess depressive symptoms, participants completed the nine-item Patient Health Questionnaire (PHQ-9) [11]. Participants indicated how frequently they experienced depressive symptoms over the past 2 weeks on a scale from 0 *not at all* to 3 *nearly every day.* The total score range is 0–27 and is interpreted as normal (0–4), mild (5–9), moderate (10–14), and severe (15–21) depression. As recommended, a PHQ-9 total score of 8 points or greater was defined as the presence of depressive symptoms for the current study [12]. Cronbach’s alpha was 0.91.

#### Suicidal risk

The suicidal risk was assessed using the Risk Assessment Suicidality Scale (RASS) [13]. RASS is a brief 12-item self-report measure of suicidal risk behaviors. It has items relevant to intention, life, and history of suicide attempts, which are rated on a 0–3 Likert scale (not at all to very much), and the scores were transformed according to the standardization study [13]. Higher scores indicate greater suicidal risk. Cronbach’s alpha for scale was 0.76.

#### Data analysis

Data was analyzed using SPSS version 26.0 (IBM Corp., NY, USA). A descriptive analysis was conducted with numbers and percentages for categorical data and mean ± SD for continuous data. Chi-square test (*χ*^2^) was used to compare the categorical data including the severity of each symptom between groups (gender, phase of studies, and history of psychiatric illness). An independent sample *t* test was used to test for differences in continuous variables (mean anxiety and depressive scores as well as suicidal risk) between groups. The significance level was set as ≤ 0.05.

## Results

### Demographic characteristics

In the current study sample of 1100 medical students, 756 (69%) were female and 344 (31.3%) were male. The mean age was 23.14 years (± 6.31). Majority (86%) were residing in the cities during the pandemic. More than 1/4th (27%) of students had previous psychiatric problems. Table 1 gives further details about sample composition.

**Table 1:** Demographic characteristics of respondents (*N* = 1100)

Characteristics	No. (%)
**Age:** Mean (S.D)	23.14 (6.31)
**Gender**	
Women	756 (68.7)
Men	344 (31.3)
**Marital status**	
Single	1084 (98.6)
Married	12 (1.1)
Divorced/widowed	4 (0.4)
**Year in medical college**	
First year	259 (23.6)
Second year	187 (17)
Third year	266 (24.2)
Fourth year	182 (16.5)
Final year	206 (18.8)
**Place of residency during the pandemic**	
City	948 (86.2)
Rural/village	152 (13.9)
**H/O medical illness**	
Yes	87 (7.9)
No	1013 (92.1)
**H/O past psychiatric problem, serious enough to make you seek professional health** (more than one response was allowed due to possibility of comorbidity)	
No	801 (73)
Anxiety	200 (18.2)
Depression	174 (15.8)
Psychosis	5 (0.5)
Bipolar disorder	7 (0.6)
Others	25 (2.3)
**Currently taking any treatment for psychiatric issues**	
No	1033 (94)
Psychotherapy	33 (3)
Antipsychotics	5 (0.5)
Antidepressant	29 (2.7)
Antiepileptic/mood stabilizers	12 (1.1)
Lithium	3 (0.3)
Tranquilizers/benzodiazepines	12 (1.1)

% may be more than 100% due to multiple responses allowed in some questions

### Measures of depression and anxiety and suicidal risk

Compared to before the COVID-19 pandemic, 514 (46.7%) students agreed that their emotional state changed and got worse in relation to the appearance of anxiety and insecurity while 33 (39.4%) admitted to worsening related to the experience of sadness.

The prevalence of depressive symptoms and GAD were 48.1% and 48.6%, respectively (Table 2). The values for females, preclinical students, and those with a history of psychiatric illness were higher than males, clinical students, and those with no past psychiatric history in all scales (*P* value < 0.001). The frequency table of responses to the individual scale items on the Risk Assessment Suicidality Scale is shown in Table 3. One in 5 medical students (approximately 20%) thought that it would be better if they were dead and 8% admitted to often think of committing suicide if they have the chance during 2-week period.

**Table 2:** Prevalence of depression and anxiety among medical students during the COVID-19 outbreak (total *N* = 1100)

Variable	Gender				Phase of study			Past H/O psychiatric illness		
	Total (*N* = 1100)	Males (*N* = 344)	Females (*N* = 756)	*P* value	Preclinical (*N* = 446)	Clinical (*N* = 653)	*P* value	Past H/O psychiatric illness present (*N* = 299)	Past H/O psychiatric illness absent (*N* = 801)	*P* value
	*n (%)*	*n (%)*	*n (%)*		*n (%)*	*n (%)*		*n (%)*	*n (%)*	
**Depression**^**a**^										
Absent	571 (51.9)	209 (60.8)	362 (47.9)	0.000*	216 (48.4)	366 (56)	0.031*	99 (33.1)	472 (58.9)	0.000**
Present	529 (48.1)	135 (39.2)	394 (52.1)		230 (51.6)	298 (45.6)		200 (66.9)	329 (41.1)	
**Generalized Anxiety Disorder (GAD)**^**b**^										
Absent	565 (51.4)	215 (62.5)	350 (46.3)	0.000**	214 (48.0)	350 (53.6)	0.039*	82 (27.4)	483 (60.3)	0.000**
Present	535 (48.6)	129 (37.5)	406 (53.7)		232 (52.0)	303 (46.4)		217 (72.6)	318 (39.7)	

Abbreviations: *n* number, *GAD* generalized anxiety disorder

^a^Depressive symptoms included individuals who scored ≥ 8 points

^b^GAD was defined as individuals who scored ≥ 7 points

*P value statistically significant < 0.05

**P value statistically significant < 0.001

**Table 3:** Frequency table of responses to the individual scale items on Risk Assessment Suicidality Scale

Statements	Not at all ***n (%)***	A little bit ***n (%)***	Much ***n (%)***	Very much ***n (%)***
Are you afraid that you are going to die?	503 (45.7)	418 (38)	117(10.6)	62 (5.6)
Do you ever think that it would be better if you were dead?	633 (57.5)	256(23.2)	131(11.9)	80(7.2)
Do you think that is a wonderful thing that you are alive?	161 (14.6)	280 (25.4)	296 (26.9)	363 (33)
Have you ever felt that’s not worth living?	532 (48.3)	337 (30.6)	139 (12.6)	92 (8.3)
Do you think of harming yourself physically?	830 (75.4)	168 (15.2)	63 (5.7)	39 (3.5)
Do you often think of committing suicide if you have chance?	853 (77.5)	152 (13.8)	59 (5.3)	36 (3.2)
Do you make plans concerning the method to use in order to end your life?	878 (79.8)	136 (12.3)	52 (4.7)	34 (3.1)
I am thinking of committing suicide, but I won’t do it	807 (73.3)	147 (13.3)	68 (6.1)	78 (7.1)
Do you enjoy your life?	114 (10.3)	312 (28.3)	433 (39.3)	241 (21.9)
Are your feeling tired from your life?	437 (39.7)	367 (33.3)	168 (15.2)	128 (11.6)
How much has your tendency to think about death and/ or suicide changed, compared to before the outbreak of COVID-19?^a^	634 (57.6)	252 (22.9)	151 (13.7)	60 (5.4)
	**Never**	**Once**	**2–3 times**	**Several times**
Have you ever hurt yourself in any way deliberately, during your whole life so far?	791 (71.9)	204 (18.5)	71 (6.4)	34 (3)
Have you ever attempted suicide, during your whole life so far?	953 (86.6)	90 (8.1)	35 (3.1)	22 (2)

^a^Not part of the RASS

Women had significantly higher mean scores than males on depression (11.2 ± 7.26 vs 8.78 ± 7.22), anxiety (9.22 ± 6.11 vs 7.00 ± 6.38), and suicidal risk (428.02 ± 309.52 vs 326.25 ±301.01). Being in the preclinical phase of studies and having a past psychiatric history also revealed a statistically significant difference in psychological measures of depression and anxiety. The *t* test also suggested that female students and students with a history of psychiatric illness differed from males and having no psychiatric history in terms of total RASS and intention, life, and history sub-scales (*P* < 0.001); however, no difference in suicidal risk was detected in students in different phases of medical studies (Table 4). All these three groups (females, preclinical students, and having past psychiatric history) also reported experiencing more severe symptom levels on depression while being female and past psychiatric history was significantly associated with more severe anxiety symptoms (Table 5).

**Table 4:** Mean scores and standard deviation of depression, anxiety, and its subscales during the COVID-19 outbreak in total cohort and subgroups

		Gender			Phase of study			Past H/O psychiatric illness		
		Mean (SD)			Mean (SD)			Mean (SD)		
Scale	Total score Mean (SD)	Men	Women	***P*** value	Preclinical	Clinical		Yes	No	***P*** value
**PHQ-9, depression symptoms**	10.46 (7.3)	8.78 (7.22)	11.2 (7.26)	0.005*	11.21 (7.50)	9.95 (7.18)	0.005*	13.43 (7.17)	9.35 (7.09)	0.000**
**GAD-7, anxiety symptoms**	8.52 (6.2)	7.00 (6.38)	9.22 (6.11)	0.034*	9.01 (6.51)	8.19 (6.09)	0.034*	11.83 (5.75)	7.29 (6.01)	0.000**
**Suicidal risk (RASS total score)** **(**Max score 1015)	396.20 (310.36)	326.25 (301.01)	428.02 (309.52)	0.000**	391.47 (306.25)	399.11 (313.47)	0.689	522.91 (315.87)	348.90 (294.82)	0.000**
**Intention scale** (Max score 500)	129.45 (171.29)	101.88 (158.48)	141.94 (175.49)	0.000**	133.44 (174.71)	126.77 (169.13)	0.526	184.92 (187.26)	108.74 (160.21)	0.000**
**Life scale** (Max score 390)	188.21 (111.60)	157.82 (112.76)	202 (108.35)	0.000**	183.25 (110.82)	191.36 (112.01)	0.237	235.23 (104.29)	170.66 (109.19)	0.000**
**History scale** **(**Max score 300)	78.54 (81.98)	66.56 (81.58)	83.99 (81.68)	0.000**	74.78 (77.95)	80.98 (84.59)	0.218	102.76 (86.88)	69.49 (78.23)	0.000**

Abbreviations: *PHQ-9* The 9-item Patient Health Questionnaire, *GAD-7* 7-item Generalized Anxiety Disorder, *RASS* Risk Assessment Suicidality Scale, *SD* standard deviation

*P value statistically significant <.05

**P value statistically significant <.001

**Table 5:** Severity categories of depression and anxiety in total cohort and subgroups

		Gender			Phase of study			Past H/O psychiatric illness		
		***N*** (%)			No. (%)			No. (%)		
Severity category	Total No. (%) 1100	Men (*N* = 344)	Women (*N* = 756)	***P*** value	Preclinical (*N* = 446)	Clinical (*N* = 653)		Yes (*N* = 299)	No (*N* = 801)	***P*** Value
**PHQ-9, depression symptoms**										
Normal	274 (24.9)	120 (34.9)	154 (20.4)	0.000**	93 (20.9)	181 (27.7)	0.021*	38 (12.7)	236 (29.5)	0.000**
Mild	297 (27.0)	89 (25.9)	208 (27.5)		123 (27.6)	174 (26.6)		61 (20.4)	236 (29.5)	
Moderate	209 (19.1)	58 (16.9)	152 (20.1)		82 (18.4)	127 (19.4)		65 (21.7)	145 (18.1)	
Severe	319 (29.0)	77 (22.4)	242 (32.0)		148 (33.2)	171 (26.2)		135 (45.2)	184 (23.0)	
**GAD-7, anxiety symptoms**										
Normal	367 (33.4)	171 (49.7)	196 (25.9)	0.000**	141 (31.6)	226 (34.6)	0.385	35 (11.7)	232 (41.4)	0.000**
Mild	288 (26.3)	61 (17.7)	228 (32.2)		112 (25.1)	176 (27.0)		76 (25.4)	213 (26.6)	
Moderate	222 (20.2)	52 (15.1)	170 (22.5)		93 (20.9)	129 (19.8)		82 (27.4)	140 (17.5)	
Severe	222 (20.2)	60 (17.4)	162 (21.4)		100 (22.4)	122 (18.7)		106 (35.5)	116 (14.5)	

Abbreviations: *PHQ-9* The 9-item Patient Health Questionnaire, *GAD-7* 7-item Generalized Anxiety Disorder

*P value statistically significant <.05

**P value < 0.001

## Discussion

Although the COVID-19 pandemic, like many other public health emergencies, is likely to have a significant psychological impact on all healthcare workers, to date, only a handful of studies have sought to examine medical student’s mental health in relation to COVID-19 [14, 15]. The results of this study, which to our knowledge is the first one from Pakistan, has shown that medical students are experiencing a severe level of psychological distress with more than two-third reporting a deterioration in their emotional health and well-being since the onset of the pandemic in Pakistan.

### Prevalence of anxiety and depression among medical students

Our results indicated that an alarming 48.6% and 48.1% of medical students were having anxiety and depressive symptoms during the COVID-19 outbreak. These prevalence estimates are not only much higher than recent reports of COVID-related self-reported anxiety and distress (25–27%) of medical students, but also in comparison to the reported global prevalence of depression (27.2%) and anxiety (33.8%) among medical students as well as from Pakistan [1, 2, 7–9]. Although it is no surprise that medical students in Pakistan experience a much higher level of anxiety and depression than the general population as well as age-matched peers during COVID-19, but the findings are quite alarming. Possible explanations for results may be excessive fear and anxiety in the community during the COVID outbreak along with plans for recruiting medical students in corona units in case of shortage of healthcare workers as well as lack of preparedness to shift to virtual learning. Medical students and teachers both faced challenges regarding online learning, and uncertainty regarding exams/electives, etc. may also have contributed to their symptoms.

Anxiety and depressive disorders are more likely to occur and worsen in the absence of interpersonal communication as seen during lockdowns. The present study builds on our recent work demonstrating a high prevalence of depression and anxiety among postgraduate trainees in Pakistan, which in comparison to medical students is still significantly less (48.1 and 48.6% in medical students’ vs 26.4% and 22.6% in postgraduate residents) suggesting that mental health problems are an issue affecting all levels of the medical workforce [16]. It also focuses on the need to intervene early in medical colleges to prevent an adverse long-term impact on physician’s health. Female medical students had higher psychological morbidity, in line with previous literature suggesting women as more vulnerable possibly due to cultural practices and societal attitudes especially in traditional societies like Pakistan. Apart from biological contribution, high rates of sexual and emotional abuse, low income, and less opportunities to work in their desired fields are all factors that contribute to higher rates of depression in females [8, 17]. However, one may also need to consider that while the rates reported by female students are high, their male counterparts may be reluctant in opening up about symptoms or asking for professional help due to societal attitudes. We also noted higher anxiety and depression in preclinical students which may be because of adjustment issues when medical students first enter medical colleges. Some studies like Chandavarkar et al. (2007) found that students transitioning from preclinical to clinical years displayed more anxiety and depressive symptoms, while other studies reported an increase in stress levels and psychological morbidity as students advance in their medical school years [18, 19]. On the other hand, another cross-sectional study noted that senior medical students exercised more, slept for a longer duration, and had more friends suggesting the possibility of better self-care skills and the ability to maintain balance in personal and professional lives during clinical years [20]. Systematic reviews and meta-analysis have found no statistically significant difference in depression and anxiety by gender and year of study [1, 2]. Consistent with findings of other reviews, students with preexisting mental health issues were more adversely impacted by the pandemic [21].

### Suicidal risk and suicidal ideation among medical students

Another major finding of this study is that on Risk Assessment Suicidality Scale (RASS) items, one in 5 medical students (approximately 20%) thought that it would be better if they were dead and 8% admitted to often think of committing suicide if they have the chance during a previous 2-week period. On the PHQ-9 item about suicidal ideation, 11.8% of medical students admitted to having suicidal thoughts nearly every day with a further 13.1% having such thoughts on almost half the days in the last 2 weeks. This frequency of suicidal ideation is quite higher than the 2-week frequency reported by Sobowele et al. and comparable (11.1–17.4%) to other reports [19, 21, 22]. Mean scores on the suicidal risk assessment scale were also extremely high in comparison to studies in the general population with significant association with past psychiatric history and female gender with suicidal ideation [13]. In view of the high prevalence of suicide ideation in the current study, it is clear that medical colleges need to develop robust systems for screening, prevention, and treatment of psychiatric issues and suicidal risk in our future physicians on a priority basis.

### Reluctance to seek help and strategies for combatting stigma

It was striking to see a limited number of medical students receiving psychiatric treatment despite the high rate of depressive and anxiety symptoms. Literature highlights various reasons for not seeking psychiatric help including poor insight, denial of illness, stigma of psychiatric illness, and fear of negative impact of students’ academic record and future career [22, 23] Realizing the high level of psychological morbidity among medical students, participating institutions and affiliated hospitals administration established and publicized dedicated confidential support services under the leadership of psychiatry faculty to help medical students during the pandemic situation. Regular webinars were coordinated to engage students through various institutional and medical students’ forums promoting healthy lifestyle strategies, better social interactions, and ensuring access to psychological interventions. All medical students were encouraged to seek counseling help at any time, if feeling distressed. Interventions also included fostering close contact through regular phone contact and real-time video conferencing through tele-psychiatry services. This was much appreciated as it provided a sense of safety and security at a time of great psychological distress.

### Clinical and policy implications of research

Study findings have important clinical and policy implications and serve to raise awareness that depression, anxiety, and suicidal ideation among medical students in Pakistan are prevalent and are unaddressed. There should be student wellness initiatives in medical colleges around the country to help with this dire situation. Regular reminders through official website/emails about proactive participation in self-care activities like exercise, good sleeping habits, balanced diet, engaging in relaxing enjoyable activities, and fulfilling relationships can act as a buffer against stress especially in vulnerable students. Uncertainty during the pandemic related to examinations and future career, etc. intensifies the students’ stress and anxiety, and institutions and educational leaders can mitigate this by regularly updating students about the future course of actions and plans. Provision of social support through virtual platforms and casual checking in e-mails/forums cannot be neglected as they help faculty and students to be there for one another in these challenging times without being physically present. They also allow students to reach out if needed. Clear and publicized policy by medical institutions about medical students who have a psychiatric history not to impact the medical college or residency applications in a negative way may help in reducing barriers for seeking help for mental health issues. Furthermore, the provision of confidential access to mental health professionals free of cost and preferably onsite as well as virtual access in medical colleges should be encouraged. Structured evidence-based programs as part of a formal wellness curriculum that are shown to reduce depression and anxiety like life skills training and mindfulness therapy may be considered by medical colleges in addition to the creation of student’s wellness committee and students mentoring from faculty [24, 25]. Discussion about mental health in campus by senior faculty including psychiatrists and psychologists on regular basis is likely to have a positive impact on help-seeking when students are feeling overwhelmed by stress, depression, and anxiety. These are particularly pertinent in Pakistan, where “concealing emotions” is the norm and there is a significant stigma towards seeking help from mental health professionals and is wrongly perceived by some students as a form of weakness [2, 26]. Well-coordinated campaigns at all levels to fight mental health stigma could be extremely useful.

### Limitations

The study has several limitations. These include cross-sectional design, the possibility of selective participation being web-based survey, students’ own or their family members’ COVID infection history affecting their well-being, and the possibility of reluctance of students to report depressive symptoms and suicidal thoughts leading to underreporting, despite the survey being anonymous. It is also important to recognize that data synthesized in this study is based on self-reported inventories and disadvantages associated with self-report data (like response bias, sampling bias) may exist. Non-exclusion of respondents with a psychiatric history may also contribute to selection bias. Additionally, although standardized screening instruments were used, full diagnostic interviews based on Diagnostic and Statistical Manual-5 (DSM-5) criteria were not done. Despite the limitations, the present study has many notable strengths, including a large sample from five medical colleges, data from both public and private sector medical colleges, representation of medical students across all the academic years, and use of standardized and validated tools for screening of depression, anxiety, and suicidal risk.

## Conclusion

Study findings provide valuable insight about the psychological impact of COVID-19 among medical students and the need for medical college’s administrators and leaders to take the lead in promoting help-seeking behaviors among medical students, who are feeling depressed and anxious and ensuring the provision of adequate support. There is a need for additional studies to monitor a student’s well-being over time to evaluate the long-term effects of the pandemic become evident. Future research to focus on how strongly depression and anxiety in medical colleges predicts psychiatric issues during residency and which interventions may be more helpful to improve their psychological well-being. All stakeholders need to collaborate to provide high-quality, timely, culturally sensitive, and confidential psychological services to the medical students to improve outcomes for the future physicians.

## Data Availability

The datasets that were generated during and/or analyzed during the current study are available from the corresponding author on request.

## Abbreviations

PHQ-9: The 9-item Patient Health Questionnaire
GAD-7: 7-item Generalized Anxiety Disorder
RASS: Risk Assessment Suicidality Scale
